# New genus of the subfamily Eutrombidiinae Thor, 1935 (Parasitengonina, Trombidioidea, Microtrombidiidae) associated with the adults of *Calomera alboguttata* (Klug, 1832) (Coleoptera, Carabidae)

**DOI:** 10.3897/zookeys.1286.173257

**Published:** 2026-07-23

**Authors:** Jawwad Hassan Mirza, Fahad Jaber Alatawi

**Affiliations:** 1 Department of Plant Protection, College of Food and Agriculture Sciences, King Saud University, Riyadh, 11451, Saudi Arabia Department of Plant Protection, College of Food and Agriculture Sciences, King Saud University Riyadh Saudi Arabia

**Keywords:** *
Arabium
*, *
Calomera
*, modified coxalae, parasitic mites, tiger beetle

## Abstract

The genus *Arabium***gen. nov**. (subfamily Eutrombidiinae, tribe Hexathrombiini), with the species, *A. calomerae***sp. nov**., is described based on larvae. Individuals of the new species were abundantly found attached to the second thoracic sternite of the tiger beetle, *Calomera alboguttata* (Klug, 1832), while a few were found attached to abdominal sternites and hind leg. The taxonomic position of the new genus is discussed in detail, and a diagnostic key to valid genera and species of the tribe is provided.

## Introduction

The family Microtrombidiidae Thor, 1935 (Acariformes, Trombidioidea) comprises two subfamilies, Microtrombidiinae Thor, 1935 and Eutrombidiinae Thor, 1935 (Southcott 1993). The latter subfamily was further categorized into three tribes based on the number of median dorsal shields, i.e. five in Hexathrombiini Southcott, 1993 and two in both Eutrombidiini Thor, 1935 and Milliotrombidiini Southcott, 1993 (Fain and Drugmand 1993; Southcott 1993). The larvae of the tribe Hexathrombiini are ectoparasites of coleopterans, with two exceptions: *Hoplothrombium quinquescutatum* Ewing, 1925 is reported on an oribatid mite inside the stomach of the American toad, *Anaxyrus americanus* (Holbrook, 1836) and *Hexathrombium southcotti* Zheng, 1997 has been captured from the ichneumonid wasp (Ewing 1925; Haitlinger 1994, 1997; Zheng 1997; Mayoral and Barranco 2005a; Felska et al. 2018).

There are four genera described in Hexathrombiini: *Alhamitrombium* Mayoral & Barranco, 2005 (monotypic), *Beronium* Southcott, 1986 (three species), *Hexathrombium* Cooreman, 1944 (10 species), and *Hoplothrombium* Ewing, 1925 (monotypic) (Mąkol et al. 2021). These genera can be differentiated by the number and shape of coxal setae, presence or absence of eyes, and of medial coxal seta 3*a* (Mąkol et al. 2021). There have been subsequent taxonomic revisions at the levels of subfamily, tribes, and genera by Vercammen-Grandjean (1967), Southcott (1986, 1993), Fain and Drugmand (1993), and Mąkol et al. (2021).

The family Microtrombidiidae is known from Saudi Arabia with *Microtrombidium lewisi* (Fain & Baker, 1993), *Trichotrombidium muscarum* (Riley, 1878), and an unidentified *Eutrombidium* sp. (Dabbour and Abdel-Aziz 1982; Kamran and Alatawi 2020). In the present study, numerous microtrombidiid mites were found attached to *Calomera alboguttata* (Klug, 1832) (Coleoptera, Carabidae). The diagnostic morphological characters of the collected specimens indicated the establishment of a new genus within the tribe Hexathrombiini. Subsequently, *Arabium* gen. nov. with the species, *A. calomerae* sp. nov., is described here based on larvae. The significance of the generic diagnostic characters and the taxonomic position of the new genus within the tribe Hexathrombiini are discussed in detail, and a diagnostic key to valid genera and species of the tribe is provided.

## Material and methods

**Figure 1.**
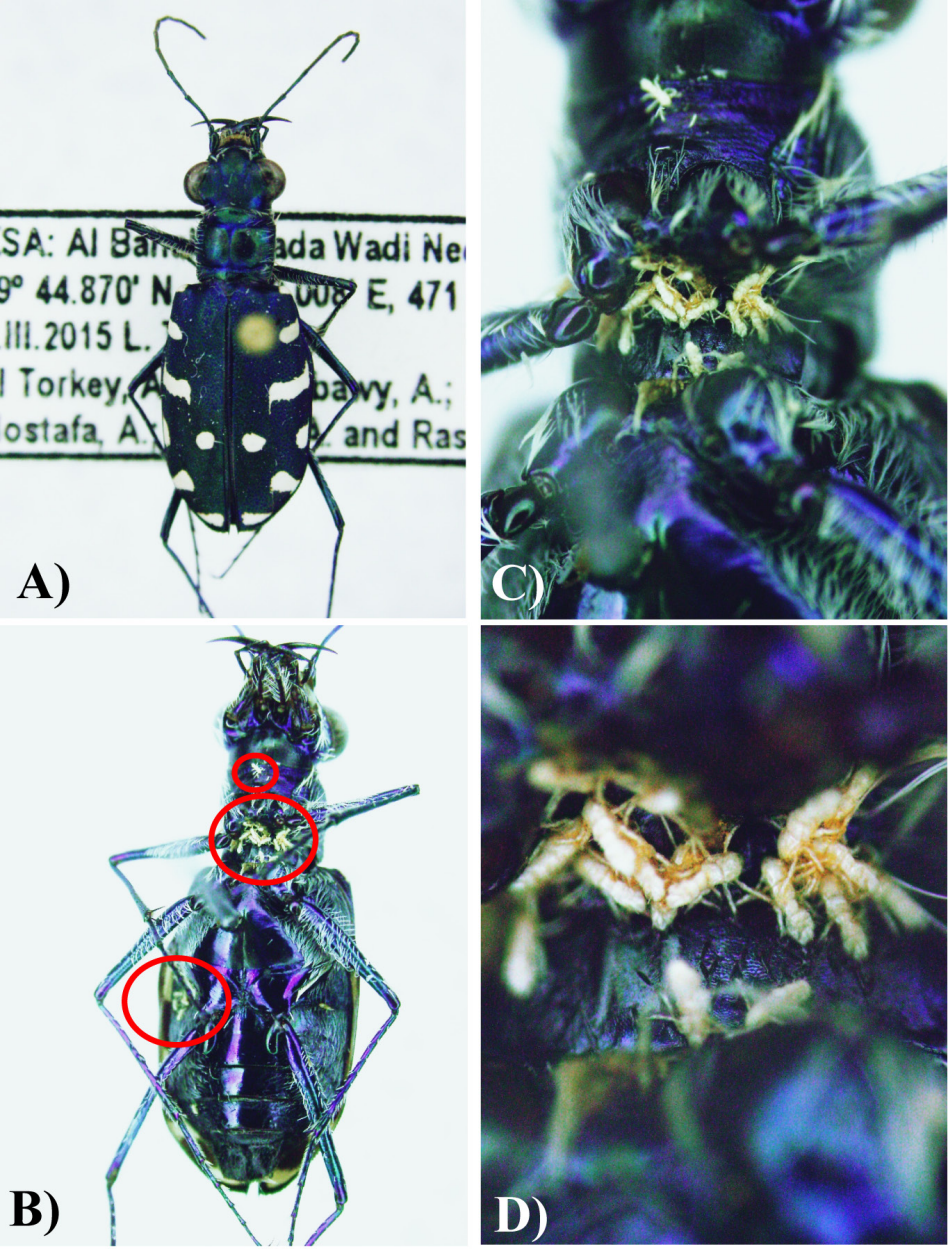
*Calomera alboguttata* (Coleoptera, Carabidae) with larvae of *Arabium calomerae* gen. et sp. nov. (Microtrombidiidae, Hexathrombiini). **A**. Dorsum of the host; **B**. Ventral habitus denoted by red circles; **C, D**. Close-up of thoracic habitus. Not to scale.

The mites were found attached to the second thoracic sternite of *Calomera alboguttata*. A few mite specimens were found clustered on the abdominal sternum and a single specimen near the neck (Fig. 1A–D). The beetle was collected using a light trap installed near a water stream in Wadi Neera, Shada, Al Baha, and identified by the expert taxonomists of King Saud University Museum of Arthropods (KSMA), King Saud University. The beetles were collected almost 10 years ago, and since then, the mite specimens have dried and hardened. The pinned specimen of tiger beetle was first submerged in lactic acid and then placed on a hot plate (40 °C) for 15 min to soften the mite bodies. The mites were then carefully detached from the beetle body with the help of a fine camel hair brush and a 000 entomological pin. The 15 detached mites were then mounted individually in Hoyer’s medium following Walter and Krantz (2009), and identified under a compound microscope (BX51, Olympus, Japan). For morphological characterization, various body parts were photographed using an auto-montage software system (Syncroscopy, Cambridge, UK) attached to a phase-contrast microscope (DM2500, Leica, Germany). Additionally, the gnathosomal features and legs were hand-drawn using a camera lucida attached to the same phase-contrast microscope. The photographs and drawings were used as templates for preparing the figures in Adobe Illustrator (Adobe Systems Inc., USA). The measurements of the holotype, as well as the mean for 14 paratypes, are given in micrometers (µm) and are presented in Table 1. The measurements of different morphological characters were taken following the system defined by Southcott (1993) for Hexathrombiini. The terminology and setal notation follow Southcott (1986, 1993), Vercammen-Grandjean (1967), and Mąkol et al. (2021). The term “setule” (adj: setulose) is used here for the setae having prominent serrations or few branches, while those with minute serrations are referred to as barbs. All specimens have been deposited in the King Saud University Museum of Arthropods (**KSMA**, Acarology section), Department of Plant Protection, College of Food and Agriculture Sciences, King Saud University, Riyadh, Saudi Arabia.

**Table 1.** Metric data (in µm) for *Arabium calomerae* gen. nov. et sp. nov. larvae.

**Character**	**Holotype**	***n***	**Mean**	**SD**	**Character**	**Holotype**	***n***	**Mean**	**SD**
IL	320	14	323	21	*c*2	26	14	26	1
IW	140	14	150	10	*c*3	17	14	21	0
IL/IW	2.3	14	2	—	*d*2	26	14	26	1
Scutum L	111	14	110	2	*d*3	22	14	23	—
Scutum W	88	14	93	3	*e*2	26	14	26	1
AM	30	14	31	1	*e*3	26	14	26	1
AMB	40	14	38	3	*f*1	26	14	26	2
MA	44	14	40	3	*f*2	28	14	28	1
AL	31	14	30	1	*f*3	29	14	29	1
AW	69	14	65	3	*h*2	54	14	55	3
PL	21	14	22	1	1*a*	28	14	27	1
PW	88	14	85	3	1*b*	11	14	11	1
AP	41	14	41	1	2*b*	8	14	9	2
SE	49	14	53	—	3*a*	20	14	20	1
SB	59	14	56	5	3*b*	8	14	8	—
SA	28	14	28	1	Cx I	52	14	51	4
SP	20	14	19	1	Tr I	28	14	26	1
ASB	88	14	85	4	Fe I	34	14	37	1
PSB	24	14	24	1	Ge I	21	14	21	1
LN	18	14	18	2	Ti I	33	14	32	1
MSA	41	14	38	2	Ta I	49	14	48	—
PSL (HS)	47	14	42	4	Leg I	217	14	205	12
PSW (LSS)	80	14	76	2	Cx II	33	14	40	1
PLN	11	14	12	2	Tr II	23	14	22	1
QL (c1)	23	14	24	2	Fe II	28	14	31	2
QW (SS)	29	14	28	3	Ge II	15	14	15	-
L3	29	14	29	1	Ti II	21	14	20	1
W3	51	14	50	1	Ta II	34	14	32	1
PLN3	15	14	15	1	Leg II	157	14	163	1
QL3 (d1)	25	14	27	1	Cx III	34	14	32	1
QW3	23	14	24	1	Tr III	23	14	23	1
L4	33	14	31	3	Fe III	36	14	33	2
W4	50	14	49	3	Ge III	16	14	16	—
PLN4	15	14	16	1	Ti III	18	14	17	—
QL4 (e1)	25	14	25	1	Ta III	18	14	17	1
QW4	24	14	25	1	Leg III	143	14	145	1
L5	34	14	38	2	SoGeI	21	14	20	0
W5	23	14	24	2		23	14	22	1
PLN5	18	14	24	3	SoTiI	16	14	16	1
QL5 (h1)	48	14	38	8		18	14	19	0
QW5	26	14	27	1		18	14	19	0
DS	25-28	14	25–28	—	SoTaI	26	14	28	1
A lens	16	14	16	—	SoGeII	40	14	40	1
P lens	13	14	14	1	SoTiII	14	14	14	—
Ocular sclerite	38	14	37	2	SoTaII	17	14	16	1
					SoGeIII	47	14	49	2

## Results and discussion

Southcott (1993) provided partial synonymy of Eutrombidiidae and associated lower taxa with the subfamily Eutrombidiinae in the family Microtrombidiidae. This classification was recognized by Gabryś (1999) in the monograph of the world Microtrombidiidae genera, and concluded in a recent molecular-based phylogenetic analysis of terrestrial Parasitengona (Costa et al. 2024). The two subfamilies were differentiated by the shape of the lateral coxalae I and hypostomala, i.e. Microtrombidiinae (lateral coxalae I unmodified, setulose, and hypostomala either modified or unmodified and setulose) and Eutrombidiinae (lateral coxalae I modified, never setulose, and hypostomala modified, never setulose) (Southcott 1993). Contrastingly, in the Manual of Acarology (Krantz and Walter 2009), Eutrombidiidae and Microtrombidiidae are considered valid without any comment on the previous synonymy by Southcott (1993), and differentiated, particularly based on the shape of coxal setae in larval stages. The concept of Southcott (1993) for the family Microtrombidiidae is followed here.

### Significant diagnostic characters for recognizing genera of Hexathrombiini

Among the three tribes of the subfamily Eutrombidiinae, the species of Hexathrombiini were described as larvae having five dorsal shields (the posteromost pygidial shield Q5 may be divided or undivided), a coxal formula of 2-2-2 or 2-1-1, and a highly modified tarsus III (Southcott 1993). This group is known only from larval stages and includes four genera, all of which have the highly modified tarsus III, with its dorsodistal projection and mop-like specialized seta, named the penicala (Southcott 1993) or lophotrix (Mąkol et al. 2021).

The diagnostic characters for recognizing genera of Hexathrombiini are provided in Table 2. Among these characters, the shape of lateral coxalae (1*b*, 2*b*, 3*b*) holds special significance. Mayoral and Barranco (2005a) distinguished *Alhamitrombium* from *Hexathrombium* and *Beronium* based on differences in the shape of lateral coxalae, in addition to the number of eye lenses and absence of seta 3*a*. This last character is debatably diagnostic of *Alhamitrombium*, as seta 3*a* is also absent in some *Hexathrombium* species. Also, determination keys to genera of Hexathrombiini by Southcott (1993), Mayoral and Barranco (2005a), and Mąkol et al. (2021) significantly used the morphological differences of lateral coxalae.

**Table 2.** Diagnostic characters among the genera of Hexathrombiini.

**Characters**	*** Alhamitrombium ***	*** Beronium ***	*** Hexathrombium ***	*** Hoplothrombium ***	***Arabium* gen. nov**.
No. of species	1	3	10	1	1
fnCx	2-1-1	1/2-1-1	2-1-1	2-2-2	2-1-1
Lateral coxala I	Bilobed	Rigid stumps/short, slight, or indistinctly bifid	Bilobed, divergent or not	Spine/spike-like, short	Spear-shaped, pointed
Lateral coxala II	Bilobed		Bilobed, divergent or not		
Lateral coxala III	Conical, round		Bilobed, divergent or not		
Medial coxal seta III	Absent	Absent	Present or absent	Present	Present
Dorsal idiosomal shields	Five	Five	Five	Five	Five
Pygidial shield (Q5)	Undivided	Undivided	Divided or undivided	Divided	Divided
Eyes	One pair	Absent	Two pairs	One pair	Two pairs
Palptibial claw	Forked	Forked	Forked	Simple	Forked
Ta III inner claw	Absent	Present or absent	Present or absent	—	Spur absent
Ta III medial claw	Falciform, simple	Falciform, simple	Falciform, simple	Falciform, simple	Falciform, heavily setulose
Reference	Mayoral and Barranco 2005a	Southcott 1986; Mąkol et al. 2021	Fain and Drugmand 1993; Southcott 1993	Grandjean 1967; Mąkol et al. 2021	Present study

Bilobed lateral coxalae 1*b*, 2*b*, and 3*b* have two morphological states in *Hexathrombium*: distinctly divergent (nine species, including the type species), or with indistinctly diverged rounded apical processes (one species) (Mąkol et al. 2021). Additionally, the setulose or simple (nude) medial coxala 1*a* has been used to differentiate *Alhamitrombium* (monotypic) from *Beronium* (three species), respectively, in the determination key to hexathrombiine genera (Mąkol et al. 2021). Medial coxal seta 3*a* are absent in both of these genera but present and always nude in *Hexathrombium* species. Furthermore, the setulose medial claw of tarsus III may represent another diagnostic feature and require deeper evaluation. The members of *Hexathrombium*, or essentially Hexathrombiini, are said to have a falciform, elongate, simple medial claw (Southcott 1993; Mąkol et al. 2021).

### Differential diagnosis of *Arabium* gen. nov.

The proposed new genus, *Arabium* gen. nov., has morphological affinity with *Hexathrombium*, based on significant diagnostic characters (Table 2). *Arabium* gen. nov. is distinguished from *Hexathrombium* by the shape of lateral coxalae 1*b*, 2*b*, and 3*b* (short spear-shaped vs divergently (distinctly or indistinctly) bilobed). This character state also distinguishes the new genus from other genera of the tribe (Table 2). Additionally, medial coxal seta 3*a* are setulose in *Arabium* gen. nov., but simple (nude) and never setulose when present in *Hexathrombium* (Table 2), and the medial claw of tarsus III is densely setulose in the new genus but simple in all other genera of Hexathrombiini.

### Taxonomy

**Family Microtrombidiidae Thor, 1935**

**Subfamily Eutrombidiinae Thor, 1935**

#### Tribe Hexathrombiini

Taxon classification

Animalia

TrombidioideaMicrotrombidiidae

Southcott, 1993

B1650B59-55F3-546B-ACBB-A24073452699

##### Type genus.

*Hexathrombium* Cooreman, 1944.

##### Diagnosis.

See Mąkol et al. 2021.

#### Arabium gen. nov.

Taxon classification

Animalia

TrombidioideaMicrotrombidiidae

11994A89-1AB2-5155-9495-402681EE8D64

https://zoobank.org/E5E8609E-0918-4FF0-B871-85EA58BBB074

##### Diagnosis.

Lateral coxalae I–III (1*b*, 2*b*, 3*b*) short, spear-shaped; medial coxal seta 3*a* with 2 or 3 setules, present very near interior border of third coxal sclerite; coxae III very close to each other; posteromost pygidial shield (Q5) distinctly divided and close to each other; setae on ventral integument posterior to coxae III densely barbed, 26 or 27.

##### Etymology.

The generic epithet refers to the name of the Arabia region from where it is reported in combination with the Latin suffix *-ium*, which is used in existing genera.

#### Arabium calomerae sp. nov.

Taxon classification

Animalia

TrombidioideaMicrotrombidiidae

8BA4EB4E-5986-5FD4-960F-41B9DF020ADE

https://zoobank.org/5B76206C-F255-45B3-B094-1B3E803C6199

##### Type material.

Larvae detached from thoracic sternites of *Calomera alboguttata*, collected by light trap. Saudi Arabia • ***holotype***: Larva; Baha • Shada, Wadi Neera; 19°44.870'N, 41°20.008'E; 03 Mar. 2015; A. El Torkey, A. El Gharbawy, A. Mostafa, A. Al Ansi, I. Rasool leg.; KSMAAS-15-Mic-Ara-H. ***Paratypes***: 14 Larvae; same data as holotype; KSMAAS-15-Mic-Ara-P1-14.

##### Diagnosis.

See diagnosis of the genus.

##### Description.

Metric data provided in Table 1.

**Larva** (*n* = 15). Idiosoma with scutum, three median dorsal shields (scutellum), and a divided posteromost pygidial shield. Scutum with strong, barbed AL and PL setae; AL larger than PL. Sensillary setae (SE) filiform. AM setae thin, simple nude, subequal to AL.

**Figures 2, 3.**
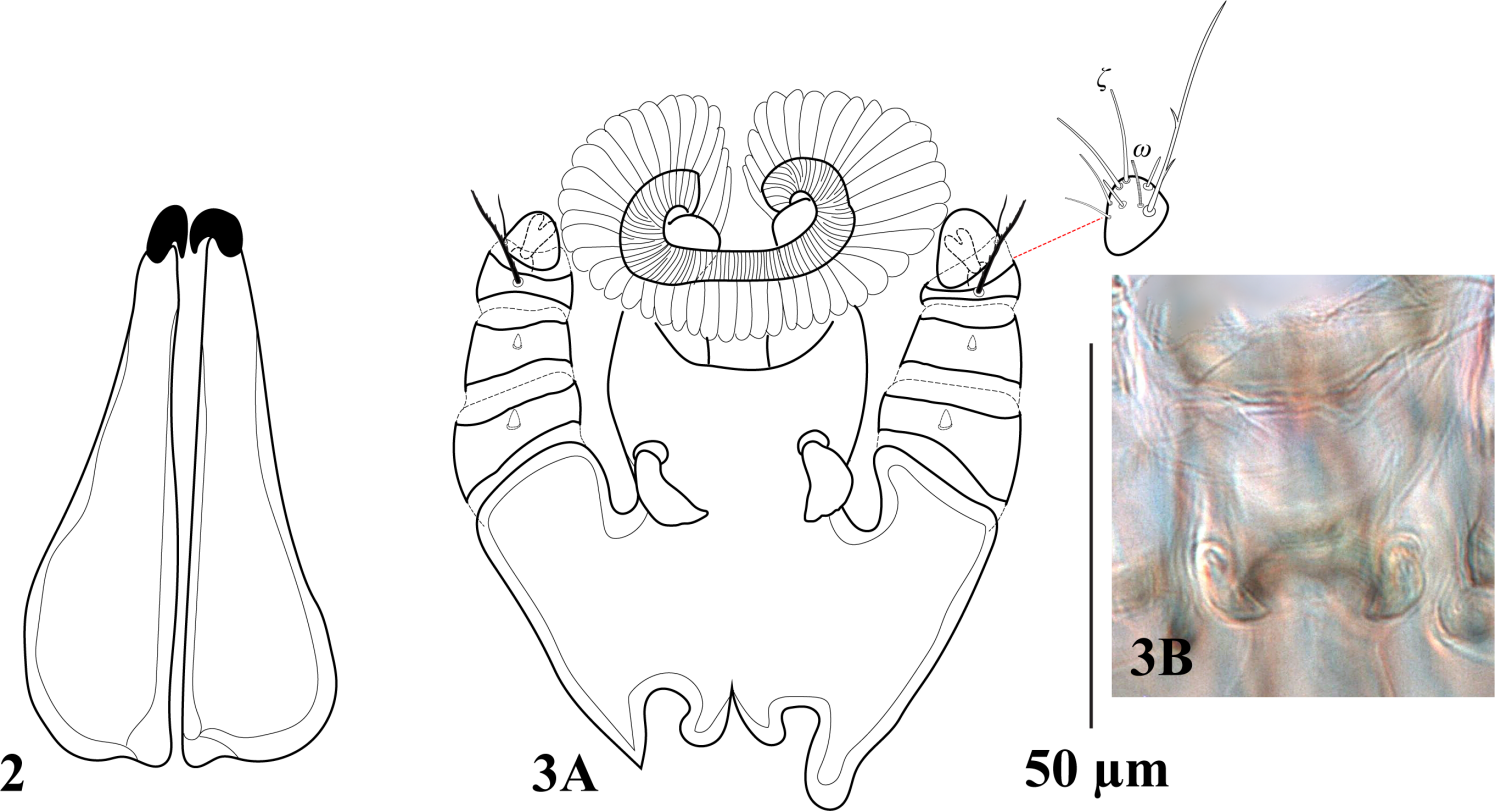
*Arabium calomerae* gen. et sp. nov. Larva. **2**. Chelicerae; **3A**. Gnathomosoma (ventral aspect); **3B**. Ventral subcapitulum.

***Gnathosoma*** (Figs 2, 3). Compact, with well-sclerotized frames of subcapitulum, chelicerae, and palps. Cheliceral claws robust, curved (Fig. 2). Stephanostome present. Internal horseshoe-shaped sclerite inserted between inner and outer cuticular sheath. Adoral setae undetectable. Subcapitular setae broad, somewhat reniform, with 2 or 3 very slight apical indentations. Palps relatively small and robust. fPp = 0-N-N-NB-BNNNNN*ωζ*. Seta on palp femur and genu short, conical. One seta (dorsal) on palp tibia simple, the second (ventral) seta long, barbed. Odontus bifid, rounded tips. The longest, most proximal seta on palp tarsus with few setules. Palpal supracoxalae (*elcp*) not detectable (Figs 3A, B).

**Figures 4–7.**
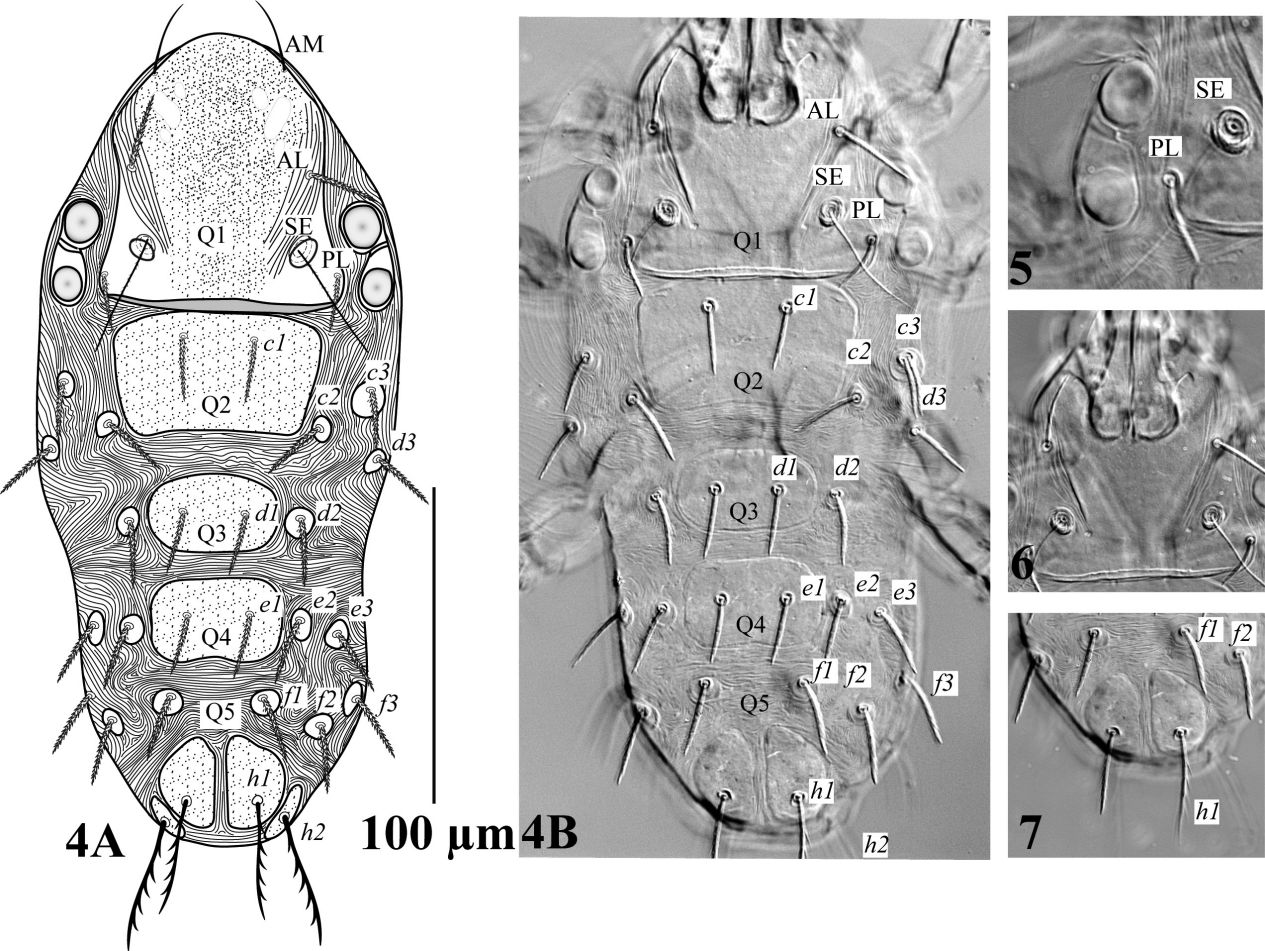
*Arabium calomerae* gen. et sp. nov. Larva. **4A, B**. Dorsum; **5**. Ocular sclerite; **6**. Scutum with weakly sclerotized regions; **7**. Divided pygidial shield.

***Dorsal idiosoma*** (Figs 4–7) oval. Integument, except for sclerites, densely striated. Scutum (Q1) (Figs 4, 5) pentagonal in outline, rounded anteriorly, bearing paired, non-sensillary setae AM, AL, and PL, and a pair of sensilla (SE). Setae AM simple, nude, subequal to PL; setae AL and PL barbed; AL longer than PL; anterolateral parts of scutum with some rare anterior longitudinal shrivels; medial part of sclerite punctate. Posterior margin almost straight, bordered with rather sclerotized band. Additionally, 2 small, semi-oval marks, probably representing less-sclerotized part of sclerite anterior to setae AL (Fig. 5). Bases of sensilla located between bases of AL and PL, closer to PL, and slightly towards medial position. AM smooth; AL and PL densely barbed and S lightly barbed along entire length; eye lenses paired (Fig. 6), each pair on a common, weakly sclerotized plate near the posterolateral margins of prodorsal scutum (Q1). Scutellum (Q2) trapezoidal, with a pair of barbed setae *c*1. Second and third scutellum (Q3, Q4) with a pair of barbed *d*1 and *e*1 setae, respectively; both sclerites broadly rectangular, with rounded corners. Setae *d*1 and *e*1 medial to anterior margin of respective scutellum. Fifth shield and pygidial shield (Q5) divided into 2 separated, lung-shaped plates, each bearing *h*1 seta (Fig. 7). fD: (2)4-(2)4-(2)4-6-(1+1)-(1+1) = 28. Setae in rows C, D, E, F, other than *c*1, *d*1, *e*1, barbed and located on small platelets; seta *c*3 duplicated, sharing same base only on left side of holotype specimen (Fig. 4b). Setae *h*1 and *h*2 longer than preceding setae, setulose, distinctly narrowed apically; *h*2 inserted in oval plates.

**Figures 8–11.**
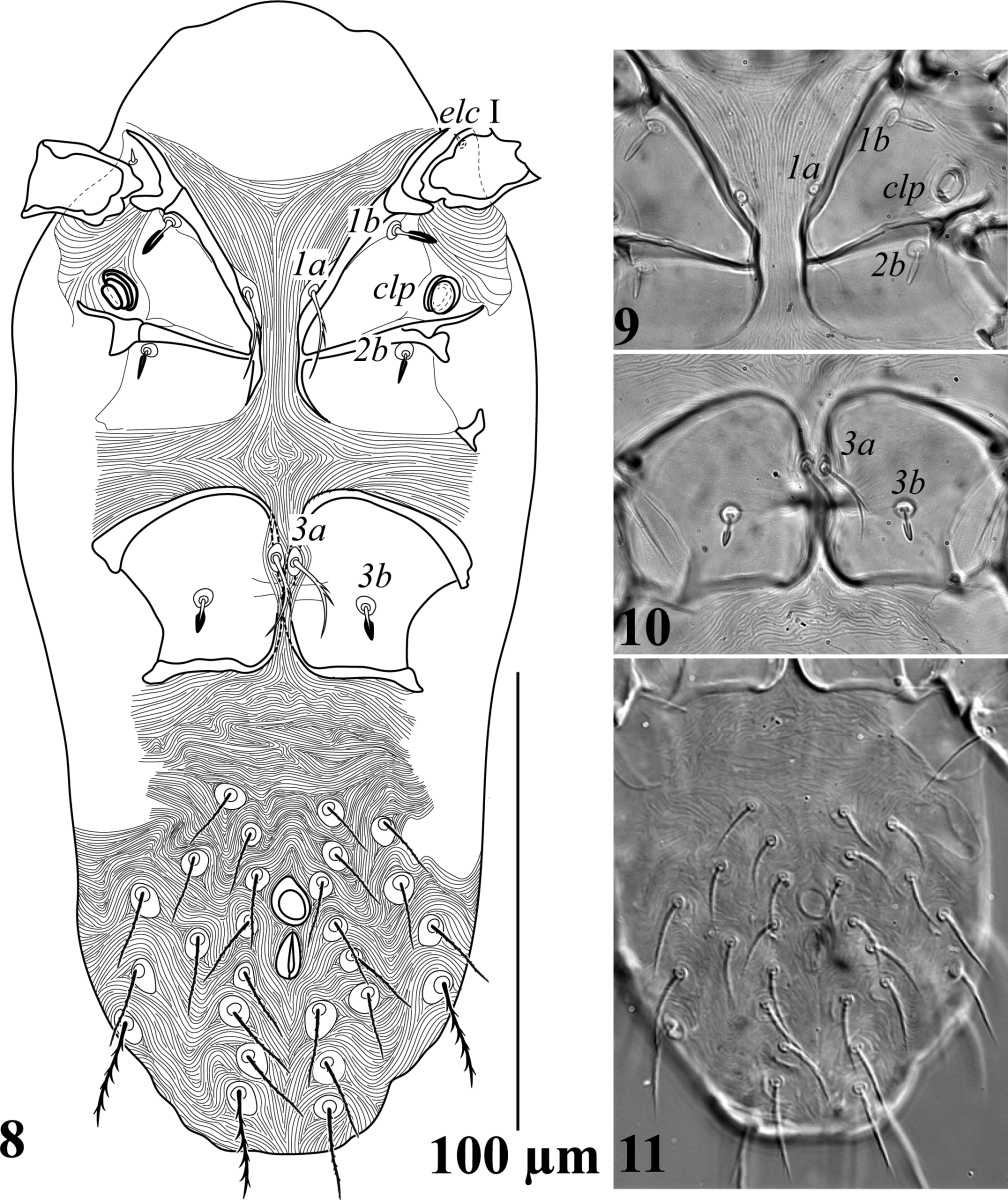
*Arabium calomerae* gen. et sp. nov. Larva. **8**. Venter; **9**. Coxae I–II; **10**. Coxa III; **11**. Anal region.

***Ventral idiosoma*** (Figs 8–11) Coxal plates (I: triangular, II: rectangular, rounded at base, III: square, with rounded corners) well sclerotized along anterior border; posterior border weakly marked or discontinuous. Anterolateral part of coxae I and III, as well as distal portion, strongly sclerotized (Figs 8, 9); sclerotization of respective parts of coxa II less pronounced. Claparède’s organs (*clp*) or urstigmata present at posterolateral corner of coxa I. Coxa I with two setae; medial coxala (1*a*) normal; 2 or 3 setules, tapering, placed on cuticular band forming medial extension of most sclerotized part of coxal frame; lateral coxala (1*b*) modified, spear-shaped (Fig. 10); coxa II with 1 modified spear-shaped lateral coxala (2*b*); coxae III very close to each other, so that medial integument bearing seta 3*a* appears raised over sclerotized border of coxa; medial coxal seta 3*a* normal, with 2 or 3 setules, placed on integument very near sclerotized border of coxa III; lateral coxala (3*b*) modified spear-shaped. Supracoxalae of coxae I (*elc* I) present. Ventral setae 26 (27 in two paratypes), tapering, densely barbed (Fig. 11). Anal opening surrounded with membraneous valves; anal sclerites absent. Pre-anal protuberance (tubercle) present, rounded, and anterior to excretory slit.

**Figures 12–15.**
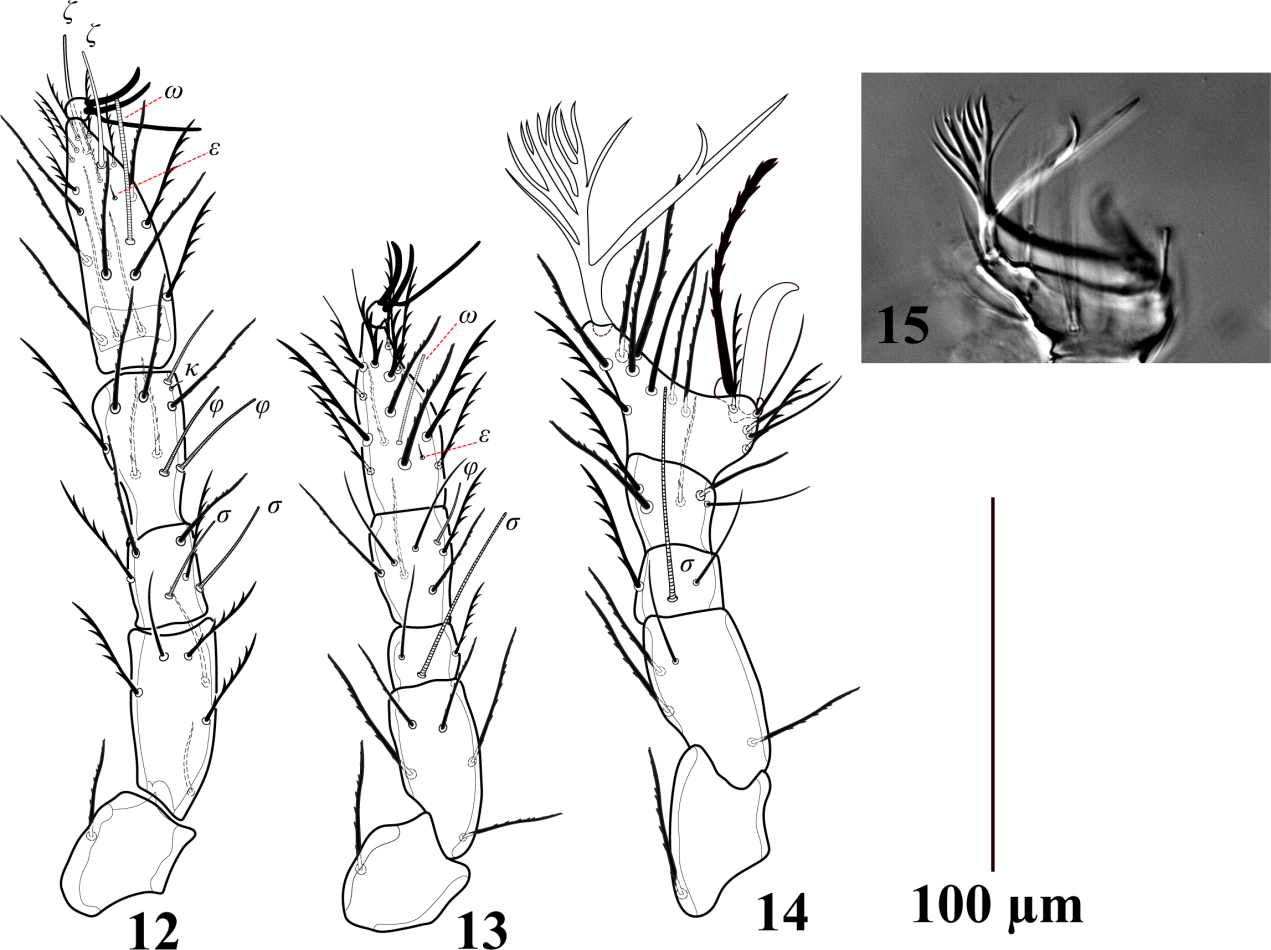
*Arabium calomerae* gen. et sp. nov. Larva. Right side of idiosoma. **12**. Leg I; **13**. Leg II; **14**. Leg III; **15**. Lophotrix on tarsus III.

***Legs*** (Figs 12–14). Segmentation formula 6-6-6. Normal setae on legs setulose to smooth. Tarsi I and II terminated with 2 claws and empodium. Claws similar in length. Empodium elongate, thin, rod-like. **Leg I** (Fig. 12). Cx with 2 setae; medial coxal setae (1*a*) slender, with 2 or 3 setules; lateral coxala (1*b*) spear shaped, Tr-1 (setulose), Fe-6 (3 setulose, 3 nude setae), Ge-6 (4 setulose, 2*σ*), Ti-10 (5 setulose, 1 nude setae, 3*φ*, 1*κ*), Ta-22 (15 setulose, 3 nude setae, 1*ω*, 2*ζ*, 1*ε*); **Leg II** (Fig. 13). Cx with 1 seta; lateral coxala (2*b*) spear-shaped, Tr-1 (setulose), Fe-5 (setulose), Ge-3 (1 setulose1 nude setae, 1*σ*), Ti-7 (4 setulose, 2 nude setae, 1*φ*), Ta-15 (13 setulose, 1*ω*, 1*ε*); **Leg III** (Fig. 14). Cx bearing 1 seta, medial coxal seta (3*a*) slender, with 2 or 3 setules, present on integument close to sclerotized border of coxa III; lateral coxala (3*b*) spear-shaped, Tr-1 (setulose), Fe-4 (3 setulose, 1 nude setae), Ge-3 (1 setulose1 nude setae, 1*σ*), Ti-5 (3 setulose, 2 nude setae), Ta-13 (9 setulose, 4 nude setae). Tarsus III highly modified at termination, with lophotrix in dorsodistal position and 2 claws located ventrodistal. Lophotrix composed of 1 branch, proximally on 1 side with 1 long secondary and tertiary sub-branches, and the main branch with several, gradually shortening, secondary and tertiary sub-branches. Inner claw robust, nude; medial claw long, falciform, densely setulose.

##### Etymology.

The specific epithet refers to the genus of tiger beetle, *Calomera*, from which the type specimens were collected.

### Key to genera and species of the tribe Hexathrombiini

Modified from Haitlinger (1994, 1997) and Mąkol et al. (2021).

**Table:** 

1	Eyes absent or reduced to ocular sclerite	***Beronium* Southcott, 1986 [2]**
–	Eyes present with one or two lenses	**4**
2	Tibia I with seven tactile setae	***B. laemostenis* Mayoral & Barranco, 2005b**
–	Tibia I with six tactile setae	**3**
3	Setae AL and PL < 55	***B. veronicae* Haitlinger, 1994**
–	Setae AL and PL > 60	***B. coiffaiti* (Beron, 1973)**
4	Eye with one lens	**5**
–	Eye with two lenses	**6**
5	fCx 2-2-2, medial coxal setae II–III (2*a*–3*a*) present, lateral coxala I (1*b*) not modified, simple	***Hoplothrombium* Ewing [ *Ho. quinquescutatum* Ewing, 1925]**
–	fCx 2-1-1, medial coxal setae II-III (2*a*–3*a*) absent, lateral coxala I (1*b*) modified, pointed, bilobed	***Alhamitrombium* Mayoral & Barranco, 2005a [ *A. tetraseta* Mayoral & Barranco, 2005a]**
6	Coxala III (3*b*) bifid, with processes diverging or adjacent to each other	***Hexathrombium* Cooreman, 1944 [7]**
–	Coxala III (3*b*) undivided, spear-shaped, apically tapering	***Arabium* gen. nov. [ *A. calomerae* sp. nov.]**
7	Pygidial shield (Q5) divided	**8**
–	Pygidial shield (Q5) entire	**13**
8	Medial coxal seta 3*a* absent	***He. sorayae* (Haitlinger, 1997)**
–	Medial coxal seta 3*a* present	**9**
9	18-20 ventral setae posterior to coxa III	***He. southcotti* Zheng, 1997**
–	Less than 18 ventral setae posterior to coxa III	**10**
10	GeI with a solenidion	**11 ^1^**
–	GeI with two solenidia	**12**
11	Scutum (Q1) large, L × W: 196–208 × 186–200; PL > 30	***He. abirami* Haitlinger, 1997**
–	Scutum (Q1) relatively small, L × W: 148–162 × 136–148; PL ≤ 30	***He. mamerti* Haitlinger, 1999**
12	Tarsus III short claw with distinct spur; leg tactile setae nude or sparsely setulose	***He. spatuliferum* Cooreman, 1944**
–	Tarsus III short claw without barbs or spur; leg tactile setae distinctly setulose	***He. fageli* Fain & Drugmand, 1993**
13	Medial coxal seta 3*a* absent	**14**
–	Medial coxal seta 3*a* present	***He. willisi* Southcott, 1993**
14	Setae AL = 32–38, PL = 14–16	***He. lubomirae* Haitlinger, 1994**
–	Setae AL = 45–52, PL = 18–22	***He. marittae* Haitlinger, 1994**

## Supplementary Material

XML Treatment for Hexathrombiini

XML Treatment for Arabium

XML Treatment for Arabium calomerae

## Footnotes

He. cicindelae Floch & Abonnenc, 1941 is not included in the key because characters to recognize it are insufficient; Mąkol et al. (2021) suggested that a redescription is needed, based on examination of type material. However, based on the leg illustrations provided by Almada and Cédola (2017) for He. cf. cicindelae, it is assumed that solenidion is absent on GeI. Based on this character, He. cf. cicindelae may be differentiated from the rest of the congeneric species at couplet 10 of the key.

## Additional information

### Artificial Intelligence (AI) use

The authors accept full responsibility for the content of the manuscript, including the disclosure of any use of AI.

No AI tools were used in the preparation of this manuscript.

### Author ORCIDs

J.H. Mirza https://orcid.org/0000-0002-1726-4331

F.J. Alatawi https://orcid.org/0000-0002-6824-2650

### Data availability

All of the data that support the findings of this study are available in the main text.
